# Mentorship and coaching to support strengthening healthcare systems: lessons learned across the five Population Health Implementation and Training partnership projects in sub-Saharan Africa

**DOI:** 10.1186/s12913-017-2656-7

**Published:** 2017-12-21

**Authors:** Anatole Manzi, Lisa R. Hirschhorn, Kenneth Sherr, Cindy Chirwa, Colin Baynes, John Koku Awoonor-Williams, the AHI PHIT Partnership Collaborative, Ahmed Hingora, Dominic Mboya, Amon Exavery, Kassimu Tani, Fatuma Manzi, Senga Pemba, James Phillips, Almamy Malick Kante, Kate Ramsey, Colin Baynes, John Koku Awoonor-Williams, Ayaga Bawah, Belinda Afriyie Nimako, Nicholas Kanlisi, Elizabeth F. Jackson, Mallory C. Sheff, Pearl Kyei, Patrick O. Asuming, Adriana Biney, Roma Chilengi, Helen Ayles, Moses Mwanza, Cindy Chirwa, Jeffrey Stringer, Mary Mulenga, Dennis Musatwe, Masoso Chisala, Michael Lemba, Wilbroad Mutale, Peter Drobac, Felix Cyamatare Rwabukwisi, Lisa R. Hirschhorn, Agnes Binagwaho, Neil Gupta, Fulgence Nkikabahizi, Anatole Manzi, Jeanine Condo, Didi Bertrand Farmer, Bethany Hedt-Gauthier, Kenneth Sherr, Fatima Cuembelo, Catherine Michel, Sarah Gimbel, Bradley Wagenaar, Catherine Henley, Marina Kariaganis, João Luis Manuel, Manuel Napua, Alusio Pio

**Affiliations:** 1 Partners In Health, Kigali, Rwanda; 2 Partners In Health, 800 Boylston Street, Suite 300, Boston, MA 02199 USA; 3 College of Medicine and Health Sciences, School of Public Health, University of Rwanda, Kigali, Rwanda; 4 Feinberg School of Medicine, Northwestern University, Chicago, IL USA; 5 Department of Global Health, University of Washington, Seattle, WA USA; 6 Health Alliance International, Beira, Mozambique; 7 Primary Care and Health Systems Department, Center for Infectious Disease Research, Lusaka, Zambia; 8 Heilbrunn Department of Population and Family Health, Mailman School of Public Health, Columbia University, New York, NY USA; 9 Ifakara Health Institute, Dar es Salaam, Tanzania; 10 Policy, Planning, Monitoring and Evaluation Division, Ghana Health Service, Accra, Ghana

**Keywords:** Mentorship, Quality improvement, Coaching, Rwanda, Ghana, Tanzania, Mozambique, Zambia

## Abstract

**Background:**

Despite global efforts to increase health workforce capacity through training and guidelines, challenges remain in bridging the gap between knowledge and quality clinical practice and addressing health system deficiencies preventing health workers from providing high quality care. In many developing countries, supervision activities focus on data collection, auditing and report completion rather than catalyzing learning and supporting system quality improvement. To address this gap, mentorship and coaching interventions were implemented in projects in five African countries (Ghana, Mozambique, Rwanda, Tanzania, and Zambia) as components of health systems strengthening (HSS) strategies funded through the Doris Duke Charitable Foundation’s African Health Initiative. We report on lessons learned from a cross-country evaluation.

**Methods:**

The evaluation was designed based on a conceptual model derived from the project-specific interventions. Semi-structured interviews were administered to key informants to capture data in six categories: 1) mentorship and coaching goals, 2) selection and training of mentors and coaches, 3) integration with the existing systems, 4) monitoring and evaluation, 5) reported outcomes, and 6) challenges and successes. A review of project-published articles and technical reports from the individual projects supplemented interview information.

**Results:**

Although there was heterogeneity in the approaches to mentorship and coaching and targeted areas of the country projects, all led to improvements in core health system areas, including quality of clinical care, data-driven decision making, leadership and accountability, and staff satisfaction. Adaptation of approaches to reflect local context encouraged their adoption and improved their effectiveness and sustainability.

**Conclusion:**

We found that incorporating mentorship and coaching activities into HSS strategies was associated with improvements in quality of care and health systems, and mentorship and coaching represents an important component of HSS activities designed to improve not just coverage, but even further effective coverage, in achieving Universal Health Care.

## Background

While the lack of trained health workers in low resource settings remains a global concern [1–3], there also remains a gap in implementation of effective strategies to build their skills, knowledge and the systems needed to ensure quality of care delivery. These gaps reflect a need to identify and invest in effective approaches to better train and support health workers to deliver quality people-centered care, a core component of health systems strengthening (HSS) needed to achieve universal health care [4, 5].

Many training programs for health care workers and managers in low income countries rely on didactic teaching [6], with limited on-the-job follow-up and practical skills-building in systems thinking [7]. However, didactic training does not effectively ensure the ability to translate theoretical knowledge into practice or address system-level barriers [8, 9]. Recent studies have found that post-training supportive supervision and coaching are effective in reinforcing learning processes, improving provider and manager motivation, and improving clinical performance [8, 10–14]. However, while supervision is common in these settings, many studies have shown that these activities often do not include critical components of supportive supervision, with limited emphasis on capacity building and problem solving, focusing more on data collection, audits and overall facility assessment [15–17].

Incorporating mentoring and coaching into supervision can transform traditional supervision into a more effective intervention to improve care quality and delivery [17, 18]. Mentoring typically includes a sustained relationship and broad skills transfer from an individual with more experience in an area to a less experienced mentee to both improve performance and also support professional development and growth of the mentee [19]. Coaching, which is often included in mentoring activities, focuses more on improvement of performance to bridge the know-do gap [20].

Since 2009, the Population Health Implementation and Training (PHIT) partnership projects in five sub-Saharan African countries (Ghana, Mozambique, Tanzania, Zambia and Rwanda) have designed and implemented context-specific HSS interventions as part of the Doris Duke Charitable Foundation (DDCF) supported African Health Initiative (AHI) [21–23] designed to improve population health outcomes and disseminate knowledge on how to achieve these goals [23]. Although there were considerable differences in the overall strategies implemented by each PHIT project, quality improvement (QI) interventions were adopted to address gaps in health worker knowledge and skills and challenges to the ability of health workers to deliver high quality care [24]. Mentorship and coaching were integrated into supportive supervision, management and capacity building for data utilization and implementation research [25, 26].

Despite the growing evidence that mentoring and coaching interventions can improve quality of care and systems [21, 27–29], less is known about the challenges of effectively adapting and integrating such interventions into different health system contexts. We present the results of a cross-site evaluation of the implementation and early outcomes of the mentorship and coaching components included within the five PHIT projects, focusing on management and health care delivery. Other papers in this supplement focus on the mentoring for research capacity and data utilization [25, 30].

Our evaluation was designed to identify differences and commonalities in implementation components and pathways, successes and challenges, and to describe the implementation design and key contextual factors that informed the final design of the mentoring/coaching intervention. These results are relevant to ongoing efforts in similar settings to ensure quality of service delivery and contribute to long term goals of improving population health in sub-Saharan Africa and more widely.

## Methods

### Study setting and design

The PHIT model was a ministry of health-academic partnership-driven intervention to implement and study health systems strengthening through multidimensional support across many of the World Health Organization (WHO)’s six health systems building blocks [23]. All five country sites were characterized by human resource constraints, particularly shortfalls in skilled workers and unmet needs in universal coverage for primary health care. The original intervention designs of the five PHIT projects differed in a number of areas, but all included mentoring and coaching [21, 31–34]. Tables 1 and 2 describe the level of mentorship and coaching intervention, setting characteristics and targeted WHO health system building blocks.

**Table 1:** African Health Initiative mentorship and coaching intervention level and setting characteristics

	Ghana	Mozambique	Rwanda	Tanzania	Zambia
Intervention Catchment Population size	500,000	1,999,000	480,000	857,000	450,000
Intervention setting	Rural	Urban/Rural	Rural	Rural	Peri-urban/Rural
National population density (people per sq. km of land area)	118	35	460	59	17
Intervention health worker density at baseline (nurses/1000)	0.62	0.23	0.63	8.49	0.70
Number of intervention health facilities	156	144	24	30	42
% of deliveries with skilled attendant at birth in intervention area at baseline	54.03	65	64.6	67.9	67.9
Health system level of mentorship and coaching intervention	Province/District/Community	Province/District	District/health facility	Community	District/Health facility

**Table 2:** African Health Initiative mentorship and coaching intervention by WHO health system building blocks

Country	Health Service Delivery	Human Resources	Health Information System^a^	Medicines/Vaccines/Technology	Leadership and Governance	Health Financing
Ghana	1	2	2	2	1	2
Mozambique	2	2	1	2	1	2
Rwanda	1	1	2	2	2	No
Tanzania	1	1	No	2	2	No
Zambia	1	1	2	2	2	2

Primary and direct focus: (1), secondary or indirect (2)

Many of the PHIT projects also incorporated mentoring in research capacity building, which is described in an accompanying paper [26]

^a^including data utilization

### Evaluation framework and data collection

**Fig. 1:**
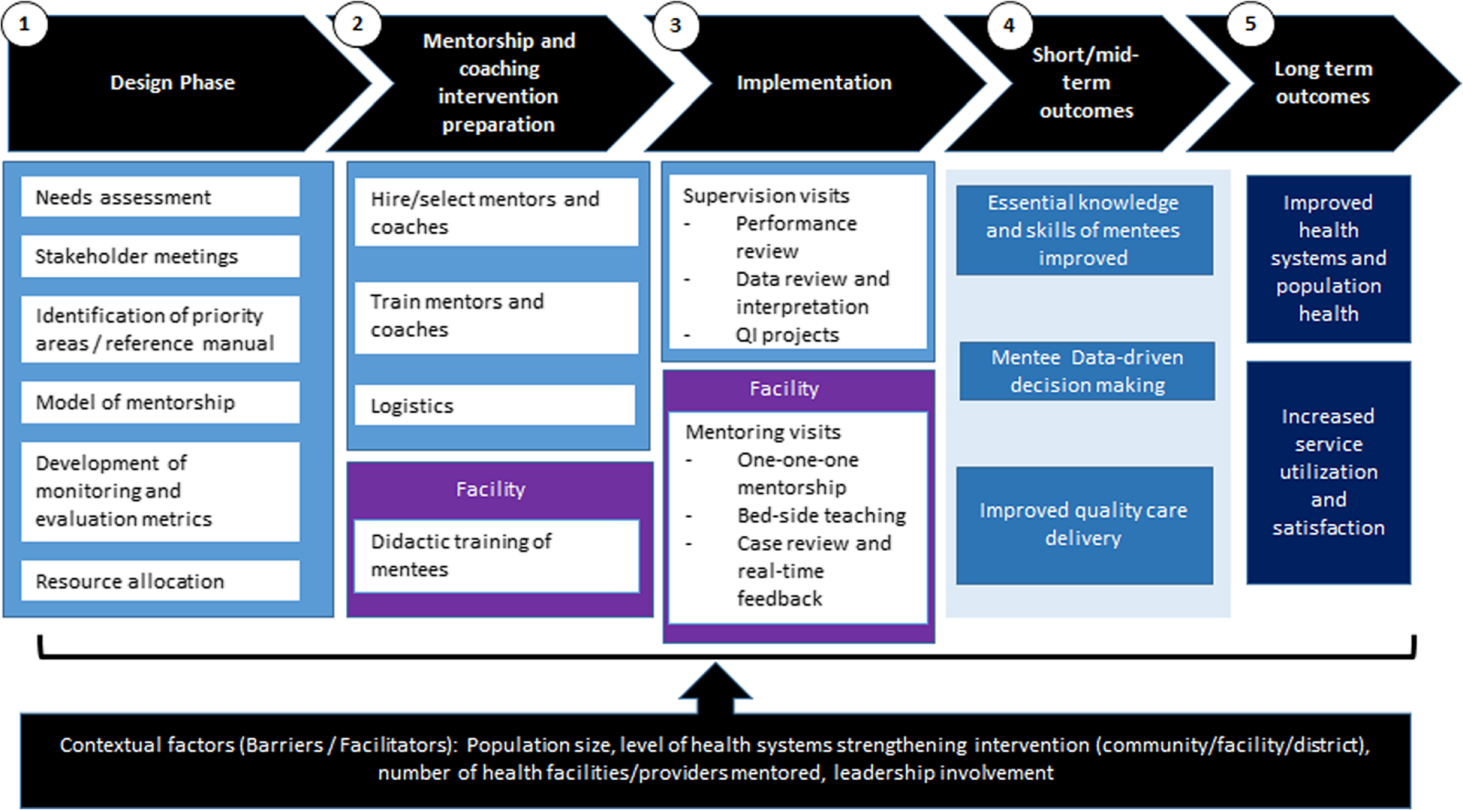
African Health Initiative mentorship and coaching: implementation and evaluation framework

We developed a framework for our analysis of the mentorship and coaching interventions reflecting the overall AHI evaluation framework [35] and the existing literature on mentorship and coaching and implementation science focusing on the implementation pathway as well as the outcomes (see Fig. 1). We used mixed methods to identify similarities and differences in the design and implementation of each project’s interventions, contextual factors influencing the design and implementation of the interventions, and explore improvements in targeted processes and shorter term outcomes. A questionnaire was designed to collect information in five categories, including: 1) mentorship and coaching goals, 2) selection and orientation of mentors and coaches, 3) integration with existing systems, 4) monitoring and evaluation, 5) challenges and successes, and 6) improvements and outcomes and contextual factors. The questionnaire was completed by key informants from each project and expanded through one-on-one semi-structured interviews. Data collected through the interviews were complemented by a review of publications from PHIT projects on mentorship and coaching and overall project qualitative and quantitative results. Follow-up telephone calls were done to augment information as needed.

### Data analysis

Results from the interviews and document review were extracted and synthesized using the conceptual framework to identify and classify emerging themes. Quantitative results from the questionnaire and document review were also extracted to provide additional information on intervention design, implementation and associated results. Coaching/mentorship outcomes included changes in mentees’ knowledge and skills, data use for decision making, and where available, changes in quality of care and management practices.

## Results

### Design phase of the PHIT mentoring and coaching interventions

Table 1 describes demographic characteristics and capacity of the intervention sites. The mentorship and coaching interventions reflected the individual country PHIT project designs, including targeted areas for improvement, local contextual factors identified through baseline needs assessment, and local challenges and culture. All of the projects focused on the subnational level (district or provincial), and four included on-site health facility work. Mozambique focused on improving management at the provincial level to ultimately improve care and health. Mentorship and coaching was designed to strengthen many of the WHO’s Health Systems Framework building blocks across all implementing sites (Table 2), although the targeted individuals and skills varied, reflecting baseline needs and intervention model.

In Rwanda and Zambia, mentorship was primarily used to strengthen quality of care delivery at health facilities and improve clinical systems of care, with a smaller focus on management and data use. In Rwanda, the Mentorship and Enhanced Supervision for Healthcare and Quality Improvement (MESH-QI) program was designed to strengthen clinical service delivery at health facilities through decentralized training of clinicians, regular supportive supervision incorporating clinical mentoring, and data collection to inform quality improvement work [27, 36, 37]. The MESH-QI program supported health center nurses in four key domains of clinical care and their supporting systems: women’s health, children under five, infectious diseases (HIV and TB), and non-communicable diseases (NCDs) (Table 3). At the district hospitals, MESH-QI targeted systems improvements [36]. Zambia implemented a facility-based supervisory intervention, in which QI teams provided clinical mentorship to health providers trained in various clinical domains [22, 38]. Community health agents were the main component of the overall PHIT intervention in Tanzania [39]. In addition to their initial training that covered key messages in maternal and child health related topics, including the community-based integrated management of childhood illness, they also received an intensive and ongoing mentorship to facilitate translation of the learned concepts into practice and improve performance.

**Table 3:** Design and preparation of African Health Initiative mentorship and coaching interventions

	Ghana	Mozambique	Rwanda	Tanzania	Zambia
Priority areas	Emergency referral, perinatal intervention, IMCI, capacity building, management	Maternal, Newborn and Child Health (MNCH), malaria, pharmacy management	MNCH care, Integrated Management of Adult and Adolescent Illness (IMAI), HIV, Noncommunicable Diseases (NCD), QI, data utilization	Training and curriculum, supervision checklist	IMAI, IMCI, Emergency Obstetric and Neonatal Care (EmONC), HIV, mentorship, leadership
Method of measuring performance	Mortality metrics, fertility rates, facility surveys	Standardized performance review matrices, observation, supervision guides	Observation checklist, Facility surveys	Case management observation tool, interviews	Chart reviews, observation tools, electronic medical record reports
Indicators	Service utilization, QI indicators, leadership management	Service utilization for MNCH and malaria services, pharmacy management	Quality of MNCH, HIV, IMAI, NCD care compared to clinical guidelines, knowledge assessment	Quality of c-IMCI service provision compared to clinical guidelines, training evaluation	Service utilization and quality of IMAI, IMCI, HIV services compared to clinical guidelines
Mentors/Coaches	Senior/experienced public health officials and clinical practitioners identified prior to intervention	Public health officials and nurses with 10 to over 25 years of experience working in, or supporting, provincial teams identified prior to intervention	Nurses and midwives with specialized skills hired at the district hospital as part of intervention	CHW supervisors in village, facility managers hired as part of intervention with at least 2 years of clinical training	Clinical officers, nurses/midwives, pharmacy technologists hired as part of intervention
Mentor training	Used Ghana’s national Leadership Development Program (LDP) to build leadership capacity in budget management and resource allocation [43]	Iterative 2-day cycles, repeated on average every 6 months, with supervision visits in between meetings Data-driven identification of areas for improvement in service provision; development and implementation of action plans to address weaknesses	Initial workshop in clinical mentorship and QI, didactic training in area of focus, ongoing supervision by mentor supervisor and clinical supervisors	Week long session for training and curriculum, and field visits to WAJA in field practicum to test and finalize supervision checklists	Mentors were trained in basic clinical packages, and were coached by experts from the University of Alabama to enhance their clinical skills (such as physical examination, ordering and interpretation of lab tests, and differential diagnosis).
Recipients of mentorship and/or coaching intervention	Community Health Officers (CHO)	Health system managers, principally at the district and facility levels	Health Center Nurses and Managers	Community Health Workers (WAJA)	Nurses, clinical officers, environmental health technologists, program officers, CHW, TBA, clinic support workers
Didactic training for recipients of mentorship and coaching intervention	18-month pre-service training and 6 months for Community Health Officers	In-service trainings based on MOH training, curriculum on using data for decision-making, linking service utilization patterns to resource planning, evaluating small-scale service delivery	Ensure mentees at the health center are trained in standard MOH packages (HIV care, EmONC, IMCI, NCDs, Essential Newborn Care)	Family planning education, supply chain management STI/HIV prevention education, safe motherhood and essential newborn care counseling and c-IMCI,	Month-long: Week 1 & 2: diagnosis and management of clinical presentations, clinical protocols Week 3: Patient registration and triage, clinical forms, data entry, medical record keeping Week 4: Same as 3 + antenatal care, postnatal care, danger signs assessment

Ghana and Mozambique focused more on improving management skills, leadership and governance at the community, facility, district or provincial level. In Ghana, coaching interventions focused on community and district leadership and governance as a strategy to build sustainable improvement in maternal and child health services, management and service capabilities at district-level [31]. The mentorship and coaching interventions were implemented using senior officers with expertise in clinical and health management from the public sector. The Mozambique PHIT project focused on improving management and leadership at the provincial and district level, strengthening existing health management units to strengthen systems and care designed to improve population health. Mentoring included management and improving use of health information systems and data [21]. PHIT project staff served as advisors with substantial experience working in, and supporting, the health system in the province. Provincial staff were composed primarily of physicians and nurses with over 5 years of experience leading provincial teams in their areas.

Despite differences in context and PHIT intervention design, there were a number of common features across sites. All sites focused on improving some of the same health service delivery areas, including maternal and child health and HIV (Table 3). While the level of intensity varied, all of the mentoring and coaching interventions included some work to increase management capacity and use of routine data to identify gaps and prioritize interventions [33, 37, 40]. Data review was also a component across the projects to guide the decision making of the mentors/coaches from the individual mentor-mentee level, to systems-wide levels [24].

### Preparation and implementation of mentorship and coaching interventions

The preparation and implementation of mentorship and coaching involved four core components: 1) mentor selection and orientation, 2) strategic deployment of mentorship and coaching teams, 3) data use for routine monitoring and supervision, and 4) on-site mentorship visits.

### Selection and orientation of mentors and coaches

The choice of mentors or coaches reflected the areas and individuals targeted for support and improvement. All coaches and mentors were experienced in the targeted area and all received an orientation and training on mentoring and data-driven coaching techniques prior to starting. Coaches and mentors who focused on facility-based care were experienced providers, while management coaching was conducted by senior managers. For example, Ghana and Mozambique used provincial and district health managers as mentors and coaches. Tanzania, focusing on community-based care, used village health workers (VHW) and health facility managers [34] to serve as mentors. In all sites, PHIT project management teams served as technical advisors and master coaches for the field-based mentors (Table 3).

### Strategic deployment of mentorship and coaching teams

The deployment of mentors or coaches was informed by site specific priorities and overall intervention design. For example, in Rwanda and Zambia, mentorship occurred during on-the-job clinical consultations, while in Mozambique and Ghana, district level meetings were used to provide coaching to provincial and district managers. Supervision visits varied by site and context. For the provincial level intervention in Mozambique, in-person visits were limited to biannual meetings to discuss performance indicators, whereas the frequency of supervision visits in Rwanda and Zambia were monthly in order to facilitate quality improvement in provider care (Table 4).

**Table 4:** Implementation of African Health Initiative mentorship and coaching intervention

	Ghana	Mozambique	Rwanda	Tanzania	Zambia
Supervisory structure for mentoring intervention	Weekly field supportive supervision, visits from regional supervisors Peer mentoring exchanges, developed supervisory approaches [42]	District performance review and enhancement meetings where health facility and district staff are supported to collate and report key performance indicators. This includes 1–2 day one-on-one meetings with facility and district staff for coaching on synthesizing and interpreting secular trends in performance indicators. Ongoing post-performance review meeting coaching via quarterly supportive supervision visits from provincial and district health systems managers, including ongoing mentorship from PHIT teams embedded in provincial health department.	After mentee’s clinical training, mentors visit each health facility every 4–6 weeks to provide mentorship in each clinical domain. Mentors conduct coaching sessions with health facility staff as needed and work with health facility leadership to address systems-gaps. Quarterly debriefing meeting to discuss quality improvement indicators.	Comprehensive training for CHW that lasts 9 months, covering biology, clinical skills. Train CHWs, provide resources for facility/supply chain at district level. Mentoring occurs through facility supervision Travel to sites monthly during first 3 months, switch to quarterly supervision afterwards.	Comprehensive training (1 month intensive on-site), on-site mentoring (month 2), monthly supervision visits by QI team (month 3 onwards) to review medical records, assess accuracy of diagnosis
Number of mentors	17	14	10	30 facility managers 50 village supervisors	18
Clinician/mentor ratio^a^	2.3	NA	12	4.8	9.3
Data use	Peer exchange, weekly clinical audit meetings [42]	Used in two-day performance meetings	Quarterly internal debriefing meetings, district data sharing meetings	Village supervisors track performance management. Used evaluation data from QoC study and 3-monthly longitudinal data system (Health and Demographic Surveillance Systems) on households	Shared through facility and national level meetings, QI team meetings
Frequency of mentorship	Monthly	Biannual	Every 4–6 weeks	Facility managers: Biannual Village supervisors: Monthly	Monthly

^a^Number of health providers on average working at health facilities divided by number of mentors in PHIT mentorship and coaching intervention

In Tanzania, a curriculum was developed and used to train community health agents, or Wawezeshaji wa Afya ja Jamii (WAJA), in key areas of health promotion, reproductive health, Integrated Management of Childhood Illness (IMCI), community-based active case findings and management. Following this training, mentorship and coaching were integrated into regular supervision in the community by the VHW mentors and in the facility by the management mentors [34].

### Data use for routine monitoring and supervision of the mentoring and coaching

All PHIT projects established data review and feedback meetings that convened at least quarterly or annually. Routinely collected data were used to inform key decision making around coaching and mentoring priorities, and feedback to key stakeholders (Table 4). At the end of each meeting, quality improvement goals were reviewed and updated as needed by participants. Recommendations were shared with appropriate management groups, such as health management committees. Subsequent mentorship and coaching visits were planned to provide technical support and facilitate implementation of these recommendations.

### On-site mentoring visits

In the PHIT projects that focused directly on facility-based care (Ghana, Rwanda, and Zambia), three main techniques were used to conduct facility-based mentoring: one-on-one mentorship, side-by-side teaching, and case reviews. The choice of techniques was informed by the mentee’s needs, workload, and the structure of clinical work. During the initial phase of the implementation where clinicians needed essential skills and competencies, side-by-side teaching was used more frequently. The more confident clinicians became, the more mentoring techniques transitioned to one-on-one mentoring. Case reviews were included to measure and improve knowledge on diagnosis and management of simpler and more complex cases. Real-time feedback between the mentor and mentee was consistently provided to reinforce best practices and identify areas for further improvement. In Mozambique, the mentoring and coaching intervention focused on improving capacity in management, leadership and accountability of health program managers at the district and provincial level [41].

### Successes

A number of successes were seen in targeted short and mid-term outcomes (Table 5). In Mozambique, mentorship and coaching interventions supported the establishment of an evidence-based Maternal and Child Health (MCH) policy, improved malaria interventions, and strengthened pharmacy management across 13 districts with 133 health facilities [21, 23]. Work with healthcare management also led to improved data quality and use to evaluate and improve programs [40]. In Rwanda, quality of under-five care, including danger signs assessment, diagnosis, and treatment improved following mentorship visits as measured in both diagnosis and recognition of danger signs [27, 37]. In Zambia, adherence to adult clinical observation guidelines improved over a 12 month period following mentorship visits [38]. In Tanzania, mentoring of the VHWs was associated with high quality Integrated Management of Childhood Illness (greater than 70% for multiple domains). In Ghana, mentoring and coaching helped accelerate effective community-based health services coverage, leading to total community-based primary health care coverage in intervention areas and improvement in childhood survival, with a 35% reduction in the under-five mortality rate [42]. There was also clear growth in a strong and visible regional and district leadership for program management and political and social engagements, which resulted in successful implementation of community-based health planning and services [43]. Improved staff satisfaction and motivation were also reported across all intervention sites. In addition to improved quality in a number of health care areas, evidence from some of the PHIT projects showed satisfaction and general acceptability of the mentoring and coaching approach [22, 38, 42, 44].

**Table 5:** Short-term outcomes following African health initiative mentorship and coaching interventions

	Improvements in Knowledge	Improvements in Quality of Service Delivery	Improvements in M&E	Improved Motivation of Health Workforce	Challenges
Ghana	Improved overall knowledge in tasks performed by Community Health Officers through observations and responses to questions	Emergency referral project - increases access to care, pushes services to community level [43]	Improved data literacy skills among health workers	Health workers invested in scaling up program [42]	Staff turnover, not strong M&E, difficult to stick to planned check-ins
Mozambique		Median data concordance improved from 56% between 2009 and 2010 (baseline period) to 87% at the end of the intervention (2012–2013) [26].	Better understanding of data, increased ownership, increased recognition of the importance of data sharing/feedback	Strong government involvement at all levels of the provincial health system, leads to more accountability and ownership, and better oversight by system managers	Low baseline computer and data analysis skills among front-line staff; conflicting priorities among limited number of provincial managers; difficulties in supporting (financially/logistically) facility and district action plans
Rwanda	Used pre/post-tests to assess knowledge changes and retention over time [district reports]	Increase in correct danger sign assessment in IMCI visits (from 47% to 99.8%) [27]. And increase in correct diagnosis from 56% to 91 [54].	Better data literacy among providers and mentors. Improvement in data quality [55]	Coaching leads to interactive, collaborative capacity building, active listening and relationships, support (not policing), real-time feedback that lead to increased motivation [55].	High demand for M&E support (data entry, analysis, reporting), difficult to stick to quarterly schedule, high turnover of health center staff, poor health facility infrastructure, logistical challenges (transport) limited mentoring time
Tanzania	Conducted evaluation of training program to identify processes that could be improved, found that correct IMCI diagnosis was satisfactory	Quality of care was ensured through measurements of correct diagnosis and treatment of under-5 illness by WAJA. 73% of 300 WAJA consultations were correctly diagnosed as measured against an IMCI-trained medical professional. 84% of 86 children diagnosed with malaria were treated correctly by WAJA.		Both clinical supervisors and WAJA cite their relationships as intrinsic motivators for better performance	Village CHW supervisors did not feel adequately compensated, tension because they were volunteers v. paid CHW. Challenges in ensuring visits to CHW from facilities.
Zambia		Improved patient-provider interaction, better outcomes, improved clinical judgement/case management, improvement in management of malaria according to protocols.	Increased use of Electronic Medical Record system, increases in data use and feedback [38].	Local ownership and collaboration, increased trust from clinical workers of QI teams, increased support for work load [38].	Shortage of qualified staff, MoH staff/volunteer attrition, poor health facility infrastructure, misunderstanding of mentor’s role by mentee, resistance to change

### Implementation challenges

A number of common challenges were encountered throughout the implementation of mentorship and coaching interventions. Turnover of both facility staff and mentors/coaches was high in the Zambia, Rwanda, and Ghana projects, resulting in needs for retraining of mentors, and difficulties in establishing critical mentor-mentee relationships, maintaining quality delivery by staff and building facility capacity to sustain improvements in systems and quality of care. Distance to health facilities, patient volume and the number of existing clinical personnel had direct impact on the design and implementation success of the clinical mentoring interventions. For example, many health facilities were located in remote geographic locations that required mentors to spend a long time travelling, resulting in transportation becoming a common barrier across PHIT projects. In many cases, mentors and coaches were required to share one vehicle with other teams of clinicians or supervisors visiting health facilities due to limited vehicle availability in order to decrease cost. Even though this was an effective strategy to efficiently use existing resources, it was a major cause of delays in mentoring activities and inhibited mentorship coverage for the full work day. Patient volume also was a challenge. A high volume of patients limited mentors’ time to provide real-time feedback and teaching moments, while low volumes, particularly in labor and delivery during mentor visit times, limited opportunities for side-by-side teaching. Competing priorities also served as a challenge, as in some cases mentors were called to work on other projects, particularly in cases where they were already embedded in Ministries of Health (MoH). This led to further decreased time for mentorship and challenges in meeting the recommended visit schedule. Finally, most projects did not have formal monitoring and evaluation (M&E) plans in place at the start to measure process and outcomes, specifically related to the mentorship and coaching components of the overall HSS intervention. Some looked mainly at outcomes (Ghana), while others had indicators more focused on facility performance (Mozambique, Zambia, and Rwanda). Low baseline data and low computer literacy among some of the mentors and coaches added a further challenge to routine data collection and effective use at the beginning and required additional training and support to ensure effective data collection, feedback and use. Table 5 summarizes the outcomes and implementation challenges.

### Contextual factors

Each PHIT project identified local contextual factors through strong engagement with local partners and a needs assessment prior to program implementation and modified their approach to reflect the needs and strengths of potential mentees. These included gaps identified in the existing skills and systems needed to achieve the projects’ HSS goals, anticipated challenges due to local environments (burden of disease, local geography), and targeted health service delivery areas. This was followed by adaption in the design of mentoring and coaching interventions and implementation strategies, and likely contributed to the success in implementation and progress in improving process and targeted outcomes. This adaption, combined with integrated monitoring and evaluation, helped overcome some of the challenges noted, including coordination of visits despite distance and transportation challenges, patient volume, and staff turnover (mentors and mentees). This integration of local context into the intervention while still maintaining the core components of effective coaching/mentoring versus simply replicating an existing model will be increasingly important as countries continue to further decentralize authority and responsibility for health system functioning and quality and outcomes of care.

## Discussion

Mentorship and coaching interventions were the core component of each of the PHIT projects, but their design and implementation were informed by identified health system needs and other contextual factors, including the population size, level of health system targeted, existing number of health facilities and providers, identified gaps being addressed, PHIT project intervention design, and level of local and regional capacity for, and progress in, health systems strengthening. For example, while Tanzania mainly focused on community health workers, other countries paid particular attention to improving healthcare delivery and processes at health facilities. We found that despite the diversity in targeted areas, mentorship and coaching were associated with improving skills, quality of management (both clinical and health systems), and contributed to the strengthening of health systems across PHIT project countries. Our findings are consistent with recent studies that have suggested mentorship and coaching as an important strategy for in-service support for skills and capacity transfer [17, 45].

A number of common and variable factors were essential for effective implementation of mentorship and coaching interventions and were critical to helping overcome challenges, which would likely be encountered in replicating the PHIT projects’ mentorship and coaching models. First, the adaptation to reflect local contextual factors including existing capacity, gaps and resources was important to design and implement an acceptable, feasible and effective intervention. This approach is consistent with best practices in implementation science, where understanding internal and external context to then adapt the intervention and implementation pathway is associated with success and increased sustainability [46, 47].

A second shared implementation component associated with success was the establishment of a strong technical advisory team of master coaches or “mentors of mentors.” In all PHIT projects, locally trained clinical or public health staff were actively involved with the delivery of the mentorship and coaching interventions, but a more senior team, including PHIT-supported staff already experienced in principles and implementation of mentorship and coaching and in the technical areas targeted, was formed. Their role was to support the initial design and implementation of the intervention (e.g. assist in the development of program and performance measurement tools, provide input into mentor/coach hiring and training). Once the intervention was underway, they provided mentors and coaches with hands-on training and capacity building in skills specific to mentoring/coaching in their targeted areas, and regular debriefing and feedback through technical mentoring to the mentors.

All PHIT projects were designed to increase the potential for intervention impact and sustainability. Consistent with other studies, they used local mentors and coaches, which is associated with more sustainable improvement of the health system and population health outcomes [48–50]. Additionally, there was active involvement of leadership at all levels of the health system, a component also associated with more effective and sustainable interventions [51]. This commitment strengthened the adoption and ownership of the mentorship and coaching programs by the local leadership and increased commitment to supporting ongoing efforts in some of the settings. Stakeholder engagement at the community, local, and national management levels was common across the PHIT projects. This engagement has been associated with increased sustainability for health care interventions in sub-Saharan Africa [52] and contributed to success and the potential for sustainability and local spread. Stakeholder engagement activities included: 1) involving local leadership and community in the intervention planning process to identify priorities for mentorship and coaching, 2) introducing regular feedback loops through community meetings and data use [22], and 3) documentation and dissemination of lessons learned to inform national policies (e.g. through attendance and presentation at local and international conferences and workshops).

All projects also established a routine monitoring and evaluation system to ensure the feedback of data to target ongoing improvement as well as improve the coaching/mentoring interventions. The availability of a measurement matrix in some programs helped prioritize measurement to drive effective implementation of mentorship and coaching activities and inform potential program adaptations needed to address ongoing or new challenges. Integration of data monitoring into mentoring and coaching was essential to inform potential priorities and enable evidence-based feedback to strengthen this component of the interventions. These routinely collected data were used to engage with stakeholders at all levels. Data were also used to inform program decision making and broader systems improvement processes, with increased data use for broad, continual quality improvement being attributed to coaching and mentoring activities [30, 40]. This may have been related to the mentoring of managers on the basics of data analysis and interpretation, integration of data with monitoring and evaluation to provide data, as well as conducting regular meetings to review data linked with improved data visibility, accountability, and evidence-based decision making and practice [26, 30].

All of the projects focused on improving the quality of service delivery and most focused on building the capacity of health management teams as an important mediating factor to strengthening care delivery and quality. This reflects the cross-PHIT focus on addressing many if not all of the WHO six building blocks to strengthen the health systems and a prioritization on quality [24]. Additionally, the availability of financial and human resources remains an important factor for effective implementation of mentorship and coaching interventions at all levels of health systems, and should be taken into account in limited-resource settings seeking to replicate PHIT mentoring and coaching interventions.

This study has a number of limitations. First, data were collected from key informants who were direct or indirect managers of the mentorship and coaching intervention, and thus may have had reservations in openly sharing challenges with mentorship and coaching interventions. Our ability to link directly to outcomes was limited by study design and absence of comparable quality of care data, when applicable, which prevented a more quantitative cross-site analysis. Furthermore, we reported quantitative results from uncontrolled pre-post evaluations. This study design limits our ability to conclude about attribution of the intervention to the changes observed. However, the deep engagement of the researchers and implementers in the targeted areas ensured that no other unknown interventions were implemented during the time period described. Despite the differences in geographic locations, levels of health systems, and resources, all sites reported improvements in targeted health service delivery areas. These findings suggest mentorship and coaching as effective interventions in various settings, but ensuring that the implementation design of this approach will improve quality will likely require adaption to reflect the context of the planned replication.

Most of the projects focused on the process of health care and health systems, and measures of experiential quality and core components of primary healthcare, including continuity and coordination, which were not routinely reported. However, further analyses in many of the projects are underway to more directly evaluate the effect of mentorship and coaching on patient outcomes and patient satisfaction and to measure the cost-effectiveness of mentoring and coaching interventions.

## Conclusion

We found that when adapted to reflect local challenges and capacity, mentorship and coaching can catalyze improvement processes to strengthen clinical practice and health systems. Critical to all of the interventions was a strategy that combined local adaptation, active involvement of local leadership and other stakeholders from the start of the design and throughout implementation, building local capacity, and integrating strong monitoring and data feedback for effective implementation and sustainability of the mentoring and coaching interventions.

While lessons learned highlight mentoring and coaching as a health systems strengthening approach, attention to ensuring that local contexts are effectively assessed to adapt intervention components as well as the implementation pathway will remain critical to successful spread. The results of cost-effectiveness studies will also help inform implementers and policy makers on the resources required to successfully replicate in other resource-limited settings. Future studies are also needed to assess the effect of mentoring and coaching on staff motivation and experiential quality, coordination, continuity, comprehensiveness, and retention in care. These systems and patient-reported outcomes are all components critical to ensuring that people-centered primary healthcare is available to everyone, a necessary step to achieve the effective quality universal health care required to meet the health-related United Nations Sustainable Development Goals [53].

## Abbreviations

AHI: African Health Initiative
DDCF: Doris Duke Charitable Foundation
HSS: Health systems strengthening
IMCI: Integrated Management of Childhood Illness
LMICs: Low- and middle income- countries
M&E: Monitoring and evaluation
MCH: Maternal and child health
MESH-QI: Mentorship and Enhanced Supervision for Healthcare and Quality Improvement
MoH: Ministries of Health
NCDs: non-communicable diseases
PHIT: Population Health Implementation and Training partnerships
QI: Quality improvement
VHW: Village Health Worker
WAJA: Wawezeshaji wa Afya ja Jamii
WHO: World Health Organization
